# Metabolic Syndrome and the Risk of Breast Cancer and Subtypes by Race, Menopause and BMI

**DOI:** 10.3390/cancers10090299

**Published:** 2018-09-01

**Authors:** Daniel T. Dibaba, Dejana Braithwaite, Tomi Akinyemiju

**Affiliations:** 1 Department of Epidemiology, University of Kentucky, Lexington, KY 40508, USA; daniel.dibaba@uky.edu; 2 Markey Cancer Center, University of Kentucky, Lexington, KY 40536, USA; 3 Department of Oncology, Georgetown University, Washington, DC 20015, USA; dejana.braithwaite@georgetown.edu

**Keywords:** metabolic syndrome, breast cancer incidence, obesity, metabolic health, NIH-AARP

## Abstract

The objective of this study was to investigate the association of metabolic syndrome (MetS) with the risk of invasive breast cancer and molecular subtypes across race, menopause, and body mass index (BMI) groups. We examined the association of metabolic syndrome and its components with risk of invasive breast cancer among 94,555 female participants of the National Institute of Health-American Association of Retired Persons (NIH-AARP) Diet and Health Study, accounting for ductal carcinoma in situ as a competing risk. Cox proportional hazard regression with the Fine and Gray method was used to generate hazard ratios (HR) and 95% confidence intervals (CI) adjusting for baseline sociodemographic, behavioral, and clinical covariates. During a mean follow-up of 14 years, 5380 (5.7%) women developed breast cancer. Overall, MetS at baseline was associated with a 13% increased risk of breast cancer compared to women without MetS (HR: 1.13, 95% CI: 1.00, 1.27); similar estimates were obtained among postmenopausal women (HR: 1.14, 95% CI: 1.01, 1.29). MetS was associated with a slight but non-significantly increased risk of breast cancer among those with both normal weight and overweight/obesity, and those with estrogen receptor positive breast cancer subtype. In the NIH-AARP cohort, MetS was associated with an increased risk of breast cancer. Further studies are needed to definitively evaluate the association of MetS with triple negative breast cancer subtypes across all levels of BMI.

## 1. Introduction 

Breast cancer remains the most common cancer type among women globally, with an estimated 266,120 new cases of invasive breast cancer diagnosed in the U.S. in 2018 [1,2,3]. Epidemiological studies indicate that although black women in the U.S. have a relatively lower incidence of breast cancer compared with white women, they experience significantly worse prognosis, including a higher risk of aggressive breast cancer subtypes and higher mortality [4,5]. Treatment strategies for breast cancer have improved significantly in the past few decades, but primary prevention strategies to reduce the incidence of breast cancer have been limited by inconsistent information regarding the role of modifiable risk factors, such as obesity and metabolic syndrome (MetS). MetS is a cluster of interrelated abnormalities that include central obesity, insulin resistance, dyslipidemia, and hypertension. Individual components of MetS, specifically central obesity [6,7], diabetes [8], and hypertension [9,10], have been associated with increased risk of breast cancer in prior studies, although the underlying biological mechanism remain unclear. Research also indicates an independent positive association between MetS and breast cancer incidence [11,12,13], aggressive cancer phenotypes [14,15,16], and distant metastasis [17]. However, it is currently accepted that breast cancer consists of several biologically distinct subtypes with potentially distinct etiology. For instance, among premenopausal women, obesity is associated with lower risk of hormone receptor-positive (estrogen receptor (ER)+ and progesterone receptor (PR)+) breast cancer, but higher risk of hormone receptor-negative breast cancer [18,19]; whereas among postmenopausal women, obesity is associated with higher risk of hormone receptor-positive breast cancer [20]. Most prior studies evaluating the association between MetS and breast cancer risk are based on case-control studies with limited racial diversity and sample size, and evidence from prospective studies in the U.S. is limited. Given the increasing epidemic of obesity and associated metabolic conditions in the U.S. and globally, it is critically important to determine the nature of the association between MetS and breast cancer risk by subtype, and across levels of race, menopausal status, and BMI. 

This study examines the association of MetS and its components with risk of breast cancer and subtypes by levels of body mass index (BMI), menopausal status, and race in a prospective cohort of older women after adjusting for sociodemographic, behavioral, and clinical covariates. 

## 2. Methods

### 2.1. Study Participants

**Figure 1:**
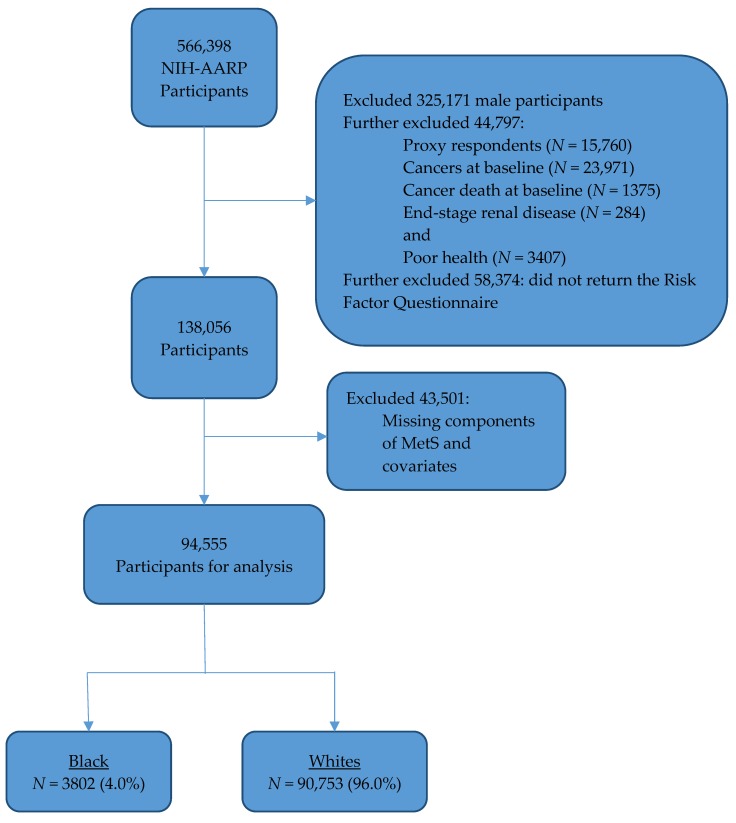
Participant selection process.

Data were obtained from the National Institute of Health–American Association of Retired Persons (NIH-AARP) Diet and Health Study. Participants in the NIH-AARP were recruited at baseline in 1995–1996 with a questionnaire mailed to 3.5 million AARP members aged 50–71 years residing in California, Florida, Louisiana, New Jersey, North Carolina, Pennsylvania, or in one of two metropolitan areas (Atlanta, Georgia; and Detroit, Michigan) in the U.S. [21]. To maximize recruitment of minority populations, smaller states and metropolitan areas with a large minority population were included. The NIH-AARP cohort has been described in greater detail elsewhere [22]. Baseline data on demographics, lifestyle, and behavioral characteristics were collected and cancer outcomes were ascertained from state cancer registries with at least 90–95% complete case ascertainment in the NIH-AARP cohort [22]. Of 566,398 participants that consented and were included in the NIH-AARP cohort, participants were excluded from the current analysis due to one or more exclusion criteria: (1) male gender, (2) self-reported cancer diagnosis at baseline, (3) self-reported poor health or end-stage renal disease (a recommended primary exclusion criteria), (4) died of cancer at baseline or data obtained from proxy respondents, (5) did not return Risk Factors Questionnaire (RFQ), and (6) missing values for covariates; leaving 94,555 participants for final analyses (Figure 1). Participants with missing values were not significantly different from those with complete data based on BMI (*p* = 0.117); however, those with missing values were older, had lower education, and were more likely to be black (*p*-values < 0.05). The study was limited to only blacks and whites, as other races (4.3% of total) did not remain in the analyses due to substantial (>65%) missing values in the components of MetS, breast cancer incidence, or covariates. The Special Studies Institutional Review Board (IRB) of the U.S. National Cancer Institute approved the NIH-AARP Diet and Health Study (protocol number: OH95CN025) [23].

### 2.2. Main Exposure 

In line with the joint harmonized criteria [24], MetS is defined as the presence of at least three components at baseline including: (1) high waist circumference (WC) >88 cm for women, (2) dyslipidemia or self-reported history of elevated cholesterol level, (3) high blood pressure or self-reported history of hypertension, and (4) self-reported history of diabetes. Systolic and diastolic blood pressure, high-density lipoprotein (HDL), triglyceride, and blood glucose were not objectively measured in the NIH-AARP Diet and Health study; however, high blood pressure (hypertension), elevated cholesterol level, and type 2 diabetes were self-reported based on whether a doctor had ever told the participant that they had the condition at baseline.

### 2.3. Main Outcome

Incident invasive breast cancer was ascertained through probabilistic linkage with the cancer registry databases from the aforementioned eight original states, and three additional states (Arizona, Nevada, and Texas) in order to capture participants who moved to those states during follow-up [25]. The linkages were based on names, address, sex, social security number, and date of birth from the baseline questionnaire. Dates of diagnosis were obtained from the cancer registries; details have been published elsewhere [23]. Censoring occurred on the date of breast cancer diagnosis, death, loss to follow-up, or 31 December 2011, whichever occurred first. 

### 2.4. Study Covariates

The analysis included baseline data on age, race (non-Hispanic whites, or non-Hispanic blacks), region (West, South, Midwest, or Northwest), BMI (kg/m^2^), and education (less than high school, high school/General Education Development (GED), some college, and ≥college). BMI was derived from weight and height variables and categorized into normal BMI (18.5 kg/m^2^ ≤ BMI < 25 kg/m^2^) and overweight or obese (BMI ≥ 25 kg/m^2^). Other baseline study covariates included behavioral characteristics such as physical activity (physical activity in the past 12 months: never, rarely, 1–3 times per month, 1–2 times per week, 3–4 times per week, 5 or more times per week, or unknown), smoking (yes/no), hormonal therapy use (never used, currently using, formerly used or unknown). Additional covariates were family history of breast cancer, ovary status (both ovaries removed, both ovaries intact, or other surgery), and hysterectomy status. 

### 2.5. Statistical Analysis

Distribution of study variables by breast cancer status was examined using the chi-squared test or Fisher’s exact test. To examine the association between MetS and risk of breast cancer adjusting for a priori specified confounders, the Cox proportional hazard regression with the Fine and Gray method was used to account for ductal carcinoma in situ as a competing risk [26]. The models were adjusted for baseline covariates including age, BMI, race, physical activity, education, smoking, region, family history of breast cancer, ovary status, current hormonal therapy use, and hysterectomy. Multicollinearity between covariates was checked using the variance inflation factor (VIF) method—all VIFs were <2, and the proportional hazard assumption was checked using the cumulative martingale residuals and Kolmogorov-type supremum test [27]. In separate models, the exposure of interest i.e., MetS (yes vs. no), each MetS component, the cumulative number of MetS components, and 4 separate combinations of three individual components were tested in relation to breast cancer incidence, comparing women with MetS or individual components to those without. Additionally, stratified analysis by BMI, race, and menopausal status were conducted, with formal tests of effect modification using the maximum likelihood ratio test and the Breslow-Day-Taron test [28]. Risk of breast cancer by hormone receptor subtypes was examined using logistic regression to compare ER− vs. ER+ subtypes (case only analysis), and to compare each subtype with non-cancer cases. Results are presented as hazard ratios or odds ratios (ORs) and 95% confidence intervals (CI). A *p*-value ≤ 0.05 was considered statistically significant, and for interactions terms, *p*-values ≤ 0.1 were considered statistically significant. All analyses were conducted using SAS 9.4 (SAS Institute Inc., Cary, NC, USA). 

## 3. Results

Among 94,555 study participants (3802 black and 90,753 white women) followed up for an average of 14 years (SD: 3.6 years), 5380 participants developed breast cancer (166 blacks and 5214 whites). Compared to participants without breast cancer (Table 1), breast cancer cases were more likely to occur in whites (97% vs. 96%), in those with a college education (38% vs. 34%), overweight or obese (BMI ≥ 25: 17% vs. 16%), current hormonal therapy users (53% vs. 47%), and more likely to have a family history of breast cancer (18% vs. 12%). 

After adjusting for baseline covariates (Table 2), MetS was associated with a marginally higher risk of overall breast cancer incidence (HR: 1.13, 95% CI: 1.00, 1.27). Having a single component of MetS was associated with a 14% increased risk of breast cancer (HR: 1.14, 95% CI: 1.03, 1.25), and the risk generally increased with the number of MetS components. The risk of breast cancer was 45% higher for participants with four components of MetS compared with none (HR: 1.45, 95% CI: 0.99, 2.13). In addition, each additional component of MetS present was associated with a 10% increased risk of breast cancer (HR: 1.10, 95% CI: 1.06, 1.14, *p* < 0.0001). There was a marginally higher but non-significant association between MetS and overall breast cancer among participants with normal BMI (HR: 1.04, 95% CI: 0.69, 1.58) and overweight/obese (HR: 1.08, 95% CI: 0.95, 1.23). However, higher number of MetS components remained associated with an increased risk of breast cancer in both groups, by 11% in those with normal BMI (HR: 1.11, 95% CI: 1.03, 1.19; *p* = 0.004) and 6% in those with overweight/obesity (HR: 1.06, 95% CI: 1.00, 1.12; *p* = 0.043). Elevated cholesterol was consistently associated with overall breast cancer risk (HR: 1.06, 95% CI: 1.01, 1.12) and across BMI groups (Table 2), in normal BMI (HR: 1.13, 95% CI: 1.04, 1.23), and overweight/obese (HR: 1.03, 95% CI: 0.96, 1.11) women. High blood pressure was associated with increased risk among overweight/obese women (HR: 1.08, 95% CI: 1.01, 1.16). No significant association was observed among black women; however, a higher number of MetS components was significantly associated with breast cancer risk among white women. 

MetS or individual components were not significantly associated with ER− compared with ER+ hormone-receptor subtype overall or among post-menopausal women in case-only analysis (Table 3), or in analysis comparing each subtype with non-cancer cases (data not shown). 

**Figure 2:**
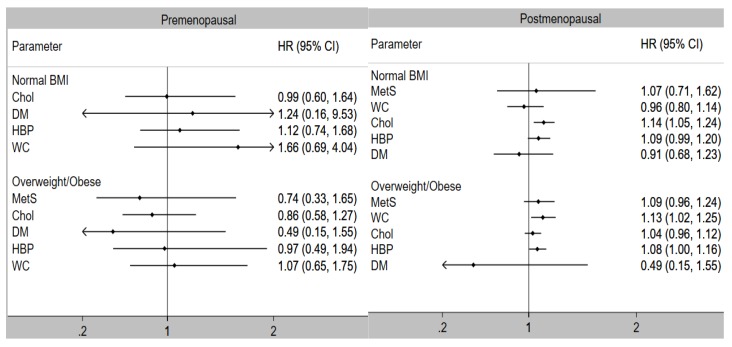
Association of metabolic syndrome (MetS) and its components with the risk of breast cancer by menopausal and obesity status. Abbreviations: BMI, body mass index; Chol, elevated cholesterol; DM, diabetes; HBP, high blood pressure; HR, hazard ratio; WC, high waist circumference. The HR for the association of MetS with breast cancer risk was non-estimable for normal BMI premenopausal women. The models are adjusted for age, race, BMI, region, physical activity, smoking, marital status, family history of breast cancer, ovary status, hysterectomy, and hormonal therapy use.

MetS was associated with higher risk of breast cancer in postmenopausal (HR: 1.14, 95% CI: 1.01, 1.29) but not in pre-menopausal (HR: 0.83, 95% CI: 0.38, 1.78) women (Table 4). High waist circumference (HR: 1.12, 95% CI: 1.04, 1.20), elevated cholesterol (HR: 1.07, 95% CI: 1.01, 1.13), and high blood pressure (HR: 1.11, 95% CI: 1.05, 1.17) were each significantly associated with higher risk of breast cancer among postmenopausal women, and there was a clear trend in increased risk with increased number of MetS components among postmenopausal women. When further stratified by BMI (Figure 2), the lack of association with MetS among pre-menopausal women remained, regardless of BMI. In postmenopausal women, elevated cholesterol (HR: 1.14, 95% CI: 1.05–1.24) remained associated with increased risk of breast cancer among women with normal BMI, whereas high waist circumference (HR: 1.13, 95% CI: 1.02, 1.25) and high blood pressure (HR: 1.08, 95% CI: 1.00–1.16) were associated with a higher risk of breast cancer in women classified as overweight/obese. 

To assess which cluster of MetS components was most strongly associated with breast cancer risk, we evaluated every possible combination compared with those without each combination in relation to overall breast cancer incidence, and observed that clusters including high waist circumference were associated with statistically non-significant higher risk, ranging from 12 to 15% (Table 5). 

## 4. Discussion 

In the large NIH-AARP prospective cohort, MetS was significantly associated with increased risk of breast cancer overall, and the risk increased as the number of MetS components present at baseline increased. These associations were observed only among post-menopausal women. Among the four distinct clusters of MetS components, all three clusters associated with a notable increased risk of breast cancer included high waist circumference and other combinations of diabetes and high blood pressure. 

Other studies observed an increased risk of breast cancer among individuals with MetS or metabolically healthy, overweight, or obese [29], and a recent meta-analysis of observational studies reported a 56% increased risk of breast cancer among those with MetS [30]. Among the five studies included in the meta-analysis, two studies [31,32] were nested case-control, whereas the other three were longitudinal [33,34,35] in design. The U.S. study included in the meta-analysis [33] was relatively small and focused on postmenopausal women in the Women’s Health Initiative clinical trial, whereas three other included studies [31,32,34] were conducted in Europe—one of them being relatively large [34] and one was conducted among the Japanese population [35]. The result of another meta-analysis of observational studies [36], two of which overlap with the previous meta-analysis [30], indicated a borderline non-significant 11% increased risk of breast cancer in postmenopausal women with MetS in cohort studies, a two-fold increased risk in studies with other study designs, and a 52% increased risk overall. Another study from a European cohort observed that MetS was associated with a lower risk of breast cancer in premenopausal women [34]. A recent cohort study among postmenopausal women in the Sister Study in the U.S. found normal-weight women with one or more components of MetS had about 26% higher risk of breast cancer [29]. Other case-control [11,37] and case-cohort studies [38] have also shown strong associations between MetS and breast cancer. Consistent with these prior studies, we observed a modestly higher risk of breast cancer among women with MetS or its individual components; however, these results were only significant among post-menopausal white women. Our study adds unique insights to this growing area of research, especially showing that MetS was associated with a marginally lower risk of ER− compared to ER+ breast cancer among American women, and highlighting the central role of high waist circumference in MetS-associated risk. A similarly higher ER+ breast cancer risk was found among overweight or obese postmenopausal women with one or more components of MetS in the Sister Study [29]. 

There are several biological mechanisms underlying this increased breast cancer risk among individuals with MetS, and studies suggest that the risk associated with multiple MetS components may be synergistic, such that the combined effect is worse than that of individual components [39,40,41]. Although we did not observe direct evidence of synergistic effects of multiple components in the present study, the biological mechanisms linking MetS components and breast cancer risk are likely interconnected. For instance, central obesity and increased adiposity in MetS likely contribute to breast cancer risk through alterations in hormonal regulation leading to over-production of estrogen and intense aromatase activity, ultimately resulting in breast tissue proliferation [42]. We observed higher breast cancer risk among post-menopausal women with normal BMI and overweight/obesity, suggesting that strategies focused on reducing central obesity (regardless of BMI) after menopause may be a key prevention strategy for breast cancer. The reduced risk of ER− breast cancer associated with MetS also suggests that obesity may lead to increased endogenous estrogen post-menopause that may contribute to tumorigenesis. Larger studies are needed to definitively evaluate this hypothesis. 

Another potential mechanism involves insulin resistance and hyperinsulinemia, which are common features of MetS. Hyperinsulinemia increases the bioavailability of insulin-like growth factor 2 (IGF2) through its effect on growth hormone in liver [42]. Both insulin resistance and IGF2 affect energy metabolism, cell differentiation and proliferation, and suppression of apoptosis [43]. Adipokine production is elevated in MetS [44,45], which has also been implicated in increased risk of breast cancer [46,47]. Other pathways include low-grade chronic inflammation [48] and cholesterol [49]. In a highly proliferative microenvironment such as breast cancer, cholesterol is required for the formation of new cell membranes [49]. It is likely that the mechanisms that regulate cholesterol uptake are altered in those with MetS, and among the three independent clusters of MetS components associated with the higher risk of breast cancer in the current study, high cholesterol was present in two. Another probable pathway is 27HC, which is a cholesterol metabolite that promotes estrogen receptor (ER)-positive breast cancer in vitro, and an ER agonist that could inhibit the liver X receptor-a regulator of cholesterol [49]. Future studies are needed to test the potential biological synergy between cholesterol and endogenous estrogen leading to increased risk of specific breast cancer subtypes. Since high cholesterol is clinically manageable, if found to play a synergetic role with central obesity and/or estrogen regulation to increase breast cancer risk, cholesterol control may be another primary prevention strategy for breast cancer among post-menopausal women.

The strength of this study includes the large sample size, which provided statistical power to detect the associations of MetS and its individual components with overall breast cancer risk and among whites. However, a limitation of the study is the lack of statistical power to detect significant differences among blacks or by BMI and menopause groups. Future studies using large dataset across cohorts, such as through large consortia like the Breast Cancer Screening Consortium, will be needed to definitively evaluate MetS in relation to breast cancer risk among racially and ethnically diverse groups and older women, and to corroborate findings across BMI levels. A second potential limitation is that while the NIH-AARP cohort included only adults aged 50–71 years, pre-menopausal women who were aged 50 years and above are likely not representative of pre-menopausal women in the general population, and may represent a distinct group of women in late pre-menopause. A third limitation of this study is that our definition of MetS relied on only four out of five components due to lack of measured biomarker data on triglycerides and HDL in the NIH-AARP cohort and those with missing values were different from those with complete data. In addition, blood pressure, cholesterol, and diabetes data were based on self-reports, which may be vulnerable to misclassification, and only measured at baseline. However, the prospective nature of the NIH-AARP data reduces the likelihood of differential misclassification, so the bias is likely to result in underestimation of the true association. The prospective design also reduced the risk of differential recall bias given that MetS components were evaluated at baseline, and participants with data collected from proxy respondents at baseline were excluded to further reduce measurement error. Nevertheless, our results are in line with other published reports in this area and add important information regarding the role of central obesity in the association between MetS and breast cancer risk in the U.S.

## 5. Conclusions 

In conclusion, MetS was associated with increased risk of breast cancer, especially among post-menopausal women regardless of obesity status, and the risk of breast cancer increased as the number of MetS components increased. Larger datasets are needed to definitively evaluate the role of MetS and individual components in risk of specific breast cancer subtypes, and in BMI and menopause stratified groups. However, based on our findings and those of others, MetS may be a useful target for lifestyle and/or clinical interventions as part of a comprehensive primary prevention strategy for breast cancer. 

## Figures and Tables

**Table 1:** Baseline characteristics of the participants by breast cancer incidence during follow-up.

Study Characteristics	Total	Breast Cancer	No Breast Cancer	*p*-Value
	*N* (%)	*N* (col%)	*N* (col%)	
**Ethnicity**				
White	90,753 (96.0)	5214 (96.9)	85,539 (95.9)	0.0003
Black	3802 (4.0)	166 (3.1)	3636 (4.1)	
**Age at entry**				
50–59	35,805 (37.9)	1899 (35.3)	33,906 (38)	<0.0001
60–69	55,638 (58.8)	3317 (61.7)	52,321 (58.7)	
70–79	3112 (3.3)	164 (3.0)	2948 (3.3)	
**Education**				
<High school	4058 (4.3)	189 (3.5)	3869 (4.3)	<0.0001
High school or GED	33,226 (35.1)	1762 (32.8)	31,464 (35.3)	
Some college	24,563 (26.0)	1401 (26)	23,162 (26)	
≥College	32,708 (34.6)	2028 (37.7)	30,680 (34.4)	
**Menopausal status**				
Premenopausal	3693 (3.9)	196 (3.6)	3497 (3.9)	0.304
Postmenopausal	90,662 (96.1)	5174 (96.4)	85,488 (96.1)	
**BMI**				
<18.5	1450 (1.5)	2344 (43.7)	39,752 (44.7)	0.047
18.5–24.9	42,096 (44.6)	1719 (32)	28,617 (32.2)	
25.0–29.9	30,336 (32.2)	776 (14.5)	12,174 (13.7)	
30.0–34.9	12,950 (13.7)	460 (8.6)	7039 (7.9)	
≥35	7499 (8.0)	556 (8.4)	6943 (7.9)	
**Metabolic syndrome**				
Yes (%)	4956 (5.2)	293 (5.4)	4663 (5.2)	0.091
**Current hormone therapy**				
Yes (%)	44,387 (46.9)	2863 (53.2)	41,524 (46.6)	<0.0001
**Family history of breast cancer**				
Yes (%)	11,949 (12.6)	951 (17.7)	10,998 (12.3)	<0.0001

Note: *p*-values were obtained from Chi-square test or Fisher exact test. Abbreviations: BMI, body mass index; col, column.

**Table 2:** Hazard ratios (HRs) ^a^ and 95% confidence intervals (CI) for metabolic syndrome (MetS) and breast cancer risk by body mass index (BMI) and race.

*N* (Events)	All *94,555* (5380) ^b^	Black 3802 (166) ^b^	White 90,753 (5214) ^b^
**Overall ^c^**			
**MetS**	**1.13 (1.00, 1.27)**	0.79(0.43, 1.43)	1.10 (0.97, 1.25)
**Components**			
High WC	**1.13 (1.05, 1.21)**	0.65 (0.42, 1.02)	**1.11 (1.02, 1.21)**
Elevated Cholesterol	**1.06 (1.01, 1.12)**	1.03 (0.76, 1.39)	**1.08 (1.02, 1.14)**
High blood pressure	**1.11 (1.04, 1.17)**	1.28 (0.92, 1.78)	**1.08 (1.02, 1.15)**
Diabetes	1.03 (0.92, 1.16)	0.83 (0.52, 1.34)	1.02 (0.90, 1.15)
**Number of MetS Components**			
0 (Ref)	1.00	1.00	1.00
1	**1.14 (1.03, 1.25)**	1.08 (0.56, 2.11)	**1.13 (1.03, 1.25)**
2	**1.26 (1.13, 1.40)**	0.78 (0.38, 1.62)	**1.25 (1.12, 1.39)**
3	**1.30 (1.12, 1.51)**	0.84 (0.36, 1.99)	**1.29 (1.10, 1.51)**
4	1.45 (0.99, 2.13)	0.51 (0.06, 4.11)	**1.48 (1.00, 2.19)**
**Normal BMI**			
**MetS**	1.04 (0.69, 1.58)	NE	1.10 (0.73, 1.67)
**Components**			
High WC	0.97 (0.82, 1.16)	NE	1.00 (0.84, 1.19)
Elevated Cholesterol	**1.13 (1.04, 1.23)**	1.12 (0.59, 2.15)	**1.13(1.04, 1.23)**
High blood pressure	1.09 (0.99, 1.20)	1.22 (0.64, 2.31)	1.09 (0.99, 1.20)
Diabetes	0.91 (0.68, 1.22)	0.28 (0.04, 1.99)	0.95 (0.71, 1.28)
**Number of MetS Components**			
0 (Ref)			
1	**1.15 (1.02, 1.29)**	0.87 (0.32, 2.33)	**1.15 (1.02, 1.29)**
2	**1.27 (1.09, 1.48)**	0.88 (0.27, 2.86)	**1.27 (1.09, 1.49)**
3	1.15 (0.75, 1.78)	NE	1.21 (0.78, 1.87)
4	1.16 (0.16, 8.40)	NE	1.47 (0.20, 10.62)
**Overweight/Obese**			
**MetS**	1.08 (0.95, 1.23)	0.83 (0.45, 1.52)	1.09 (0.96, 1.24)
**Components**			
High WC	**1.13 (1.03, 1.25)**	0.73 (0.44, 1.20)	**1.15 (1.04, 1.27)**
Elevated Cholesterol	1.03 (0.96, 1.11)	0.96 (0.67, 1.37)	1.04 (0.96, 1.12)
High blood pressure	**1.08 (1.01, 1.16)**	1.31 (0.88, 1.95)	**1.07 (1.00, 1.16)**
Diabetes	1.01 (0.89, 1.16)	0.93 (0.56, 1.55)	1.02 (0.89, 1.17)
**Number of MetS Components**			
0 (Ref)			
1	1.10 (0.92, 1.32)	1.24 (0.48, 3.24)	1.10 (0.92, 1.32)
2	1.18 (0.99, 1.41)	0.74 (0.28, 1.95)	**1.20 (1.00, 1.44)**
3	**1.24 (1.01, 1.53)**	0.95 (0.33, 2.76)	**1.25 (1.01, 1.55)**
4	1.35 (0.89, 2.05)	0.57 (0.06, 5.06)	1.40 (0.92, 2.15)

^a^ Models were adjusted for age, race (in non-race stratified models only), BMI (in non-BMI stratified models only), education, region, physical activity, smoking, marital status, family history of breast cancer, ovary status, hysterectomy, hormonal therapy use, and ovary status × BMI interaction (in non-BMI stratified models only). ^b^ *N* = number of incident breast cancers. The interaction between MetS and BMI was not significant, *p* = 0.410. Bold indicates statistically significant at the 0.05 alpha level. Abbreviations: BMI, body mass index; MetS, metabolic syndrome; NE, non-estimable; Ref, referent; WC, waist circumference. Women without MetS or respective components were the referent.

**Table 3:** Odds ratios (ORs) ^a^ and 95% confidence intervals for MetS and breast cancer risk by hormone receptor subtype ^δ^ and menopausal status (case only).

	Overall	Postmenopausal	Premenopausal
***N* (Events)**	ER− vs. ER+ 4392 (685) ^§^	ER− vs. ER+ 4212 (664)	ER− vs. ER+ 169 (21)
**MetS**	0.88 (0.60, 1.28)	0.97 (0.66, 1.43)	NE
**Components**			
High WC	0.71 (0. 57, 0.88)	0.82 (0.62, 1.08)	0.15 (0.02, 1.52)
Elevated Cholesterol	1.12 (0.95, 1.33)	1.09 (0.92, 1.29)	2.04 (0.53, 7.88)
High blood pressure	0.93 (0.78, 1.11)	0.98 (0.82, 1.17)	0.44 (0.09, 2.22)
Diabetes	0.98 (0.68, 1.41)	1.02 (0.71, 1.48)	2.92 (0.11, 78.03)
**Number of MetS Components**			
0	Ref	Ref	Ref
1	1.05 (0.78, 1.41)	1.00 (0.74, 1.34)	NE
2	0.85 (0.61, 1.18)	0.94 (0.67, 1.33)	NE
3	0.85 (0.53, 1.36)	0.99 (0.61, 1.63)	NE
4	1.18 (0.38, 3.63)	1.50 (0.48, 4.71)	NE

Adjusted covariates included age, race, BMI, marital status, family history of breast cancer, hormone therapy use, and hysterectomy. ^a^ The odds of ER- were compared to the odds of ER+. ^δ^ ER status only due to limited sample sizes for HER2 receptor status. ^§^ *N* is the overall ER− and ER+ sample size, and events are only ER− cases. Abbreviations: ER+, estrogen receptor positive; ER−, estrogen receptor negative; NE, non-estimable.

**Table 4:** Hazard ratios (HRs) ^a^ and 95% confidence intervals for MetS and breast cancer risk by menopausal status.

*N* (Events)	Postmenopausal 90,662 (5174) ^b^	Premenopausal 3693 (196) ^b^
**MetS**	**1.14 (1.01, 1.29)**	0.83 (0.38, 1.78)
**Components**		
High WC	**1.12 (1.04, 1.20)**	1.31 (0.90, 1.90)
Elevated Cholesterol	**1.07 (1.01, 1.13)**	0.88 (0.65, 1.19)
High blood pressure	**1.11 (1.05, 1.17)**	1.14 (0.82, 1.59)
Diabetes	1.05 (0.93, 1.18)	0.60 (0.22, 1.63)
**Number of MetS Components**		
0 (Ref)	1.00	1.00
1	**1.15 (1.0, 1.27)**	0.79 (0.48, 1.30)
2	**1.26 (1.14, 1.41)**	1.01 (0.59, 1.78)
3	**1.32 (1.13, 1.53)**	0.70 (0.28, 1.75)
4	1.44 (0.97, 2.12)	2.00 (0.23, 17.74)

^a^ Models were adjusted for age, race, BMI, region, physical activity, smoking, marital status, family history of breast cancer, ovary status, hysterectomy, hormonal therapy use, and ovary status × BMI interaction. ^b^ *Case* = number of incident breast cancer; *N* = sample size. Bold indicates statistically significant at the 0.05 alpha level. Women without MetS or respective components were the referent.

**Table 5:** Association between combinations of MetS components and breast cancer risk by menopausal status (HR, 95% CI).

MetS Component Combinations	Overall (*N = 94,555,* Cases = 5380) ^b^	Postmenopausal (*N* = 90,662, Cases = 5174)	Premenopausal (*N* = 3693, Cases = 196)
High blood pressure, high WC and diabetes	1.15 (0.93, 1.43)	1.11 (0.90, 1.38)	1.18 (0.28, 4.92)
Elevated cholesterol, diabetes, and high WC	1.15 (0.86, 1.53)	1.10 (0.82, 1.48)	1.14 (0.15, 8.87)
High blood pressure, diabetes and elevated cholesterol	1.04 (0.80, 1.34)	1.02 (0.79, 1.32)	0.60 (0.08, 4.42)
High blood pressure, elevated cholesterol and high WC	1.12 (0.97, 1.29)	1.09(0.94, 1.26)	0.79 (0.34, 1.79)

Models were adjusted for age, race, BMI, education, region, physical activity, smoking, marital status, family history of breast cancer, ovary status, hysterectomy, hormonal therapy use, and ovary status × BMI interaction. Women without the respective combination of the components were the referent in each analysis. ^b^ *cases* = number of incident breast cancer. Abbreviations: WC, waist circumference.
